# MRI-based machine-learning radiomics of the liver to predict liver-related events in hepatitis B virus-associated fibrosis

**DOI:** 10.1186/s41747-025-00602-0

**Published:** 2025-08-27

**Authors:** Yuankai Luo, Qinian Luo, Yaobo Wu, Shaorui Zhang, Huan Ren, Xiaofeng Wang, Xiujuan Liu, Qin Yang, Weiguo Xu, Qingsong Wu, Yong Li

**Affiliations:** 1 Hepatobiliary and Pancreatic Tumor Diagnosis and Treatment Center, Yuebei People’s Hospital, Shaoguan, China; 2 Zhuhai Interventional Medical Center, Zhuhai People’s Hospital (Zhuhai Clinical Medical College of Jinan University), Zhuhai, China; 3 Department of Pain Management, Shaoguan Zhengtong Hospital, Shaoguan, China; 4 Infection and Hepatology Department, Zhuhai Clinical Medical College of Jinan University (Zhuhai People’s Hospital), Zhuhai, China; 5 Department of Ultrasound Medicine, Zhanjiang Central People’s Hospital, Zhanjiang, China; 6 School of Medicine, Sun Yat-sen University, Shenzhen, China; 7 Department of Radiology, Zhuhai People’s Hospital (Zhuhai Clinical Medical College of Jinan University), Zhuhai, China

**Keywords:** Hepatitis B (chronic), Liver fibrosis, Machine learning, Magnetic resonance imaging, Radiomics

## Abstract

**Background:**

The onset of liver-related events (LREs) in fibrosis indicates a poor prognosis and worsens patients’ quality of life, making the prediction and early detection of LREs crucial. The aim of this study was to develop a radiomics model using liver magnetic resonance imaging (MRI) to predict LRE risk in patients undergoing antiviral treatment for chronic fibrosis caused by hepatitis B virus (HBV).

**Methods:**

Patients with HBV-associated liver fibrosis and liver stiffness measurements ≥ 10 kPa were included. Feature selection and dimensionality reduction techniques identified discriminative features from three MRI sequences. Radiomics models were built using eight machine learning techniques and evaluated for performance. Shapley additive explanation and permutation importance techniques were applied to interpret the model output.

**Results:**

A total of 222 patients aged 49 ± 10 years (mean ± standard deviation), 175 males, were evaluated, with 41 experiencing LREs. The radiomics model, incorporating 58 selected features, outperformed traditional clinical tools in prediction accuracy. Developed using a support vector machine classifier, the model achieved optimal areas under the receiver operating characteristic curves of 0.94 and 0.93 in the training and test sets, respectively, demonstrating good calibration.

**Conclusion:**

Machine learning techniques effectively predicted LREs in patients with fibrosis and HBV, offering comparable accuracy across algorithms and supporting personalized care decisions for HBV-related liver disease.

**Relevance statement:**

Radiomics models based on liver multisequence MRI can improve risk prediction and management of patients with HBV-associated chronic fibrosis. In addition, it offers valuable prognostic insights and aids in making informed clinical decisions.

**Key Points:**

- Liver-related events (LREs) are associated with poor prognosis in chronic fibrosis.
- Radiomics models could predict LREs in patients with hepatitis B-associated chronic fibrosis.
- Radiomics contributes to personalized care choices for patients with hepatitis B-associated fibrosis.

**Graphical Abstract:**

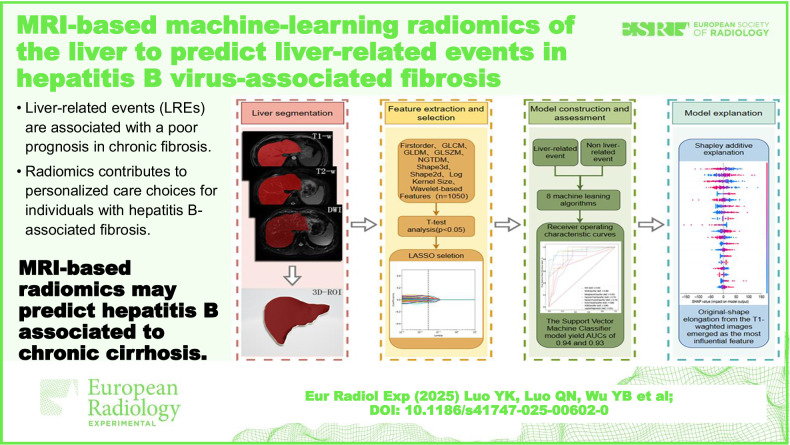

## Background

Hepatitis B virus (HBV) infection is a major global health concern [1]. About 20% of those infected develop chronic hepatitis, and 10–20% of these cases progress to cirrhosis, a progressive liver disease marked by fibrosis and impaired liver function [2]. Although oral antiviral therapy is the standard treatment for HBV-associated fibrosis [3, 4], liver-related events (LREs), such as ascites, variceal bleeding, hepatorenal syndrome, hepatic encephalopathy, and hepatocellular carcinoma, may still occur [5–8]. LREs in fibrosis indicate a poor prognosis and diminish health-related quality of life [9]. Early identification and prediction are essential for implementing interventions that improve patient outcomes.

Magnetic resonance imaging (MRI), a powerful tool for diagnosing and managing liver disease, enables accurate, noninvasive detection and characterization. Its excellent soft tissue resolution provides detailed insights into liver lesions and fibrosis staging [10, 11]. Recent advances in machine learning have enabled the analysis of quantitative imaging features, known as radiomics [12]. These features capture subtle tissue variations on MRI and hold promise for predicting treatment response and prognosis across various diseases [13, 14].

Research on liver MRI-based radiomics for predicting LREs in HBV-associated fibrosis remains limited. Most studies rely on clinical factors or serum biomarkers [15, 16], underutilizing imaging data. While radiological imaging is the only noninvasive method for capturing liver disease’s spatial heterogeneity, its full potential has yet to be realized [17]. Therefore, we employed various machine learning algorithms to develop predictive models using MRI radiomics features to identify imaging factors most associated with HBV-associated fibrosis and assess LRE risk.

## Methods

### Patient selection

This study adhered to the Declaration of Helsinki and was approved by the Ethics Committee of Zhuhai People’s Hospital (Approval Number 37 (2021)). Due to its retrospective design, informed consent was waived. All patient records and information were anonymized and de-identified prior to analysis.

This study retrospectively analyzed the data of patients with HBV who received antiviral treatment between January 2022 and July 2023. We included all consecutive patients who met the inclusion and exclusion criteria. Patient data were retrieved from the hospital’s electronic medical records. The inclusion criteria were: (1) age 18–75 years: (2) clinical diagnosis of chronic hepatitis B with hepatitis B surface antigen positivity for at least six months and HBV deoxyribonuclecic acid−DNA levels > 5 log_10_ copies/mL; (3) liver stiffness measurement (LSM) ≥ 10 kPa; and (4) current oral antiviral therapy for chronic hepatitis B. The exclusion criteria were: (1) no upper abdominal MRI before the LRE; (2) inadequate MRI quality due to artifacts, low signal-to-noise ratios, poor resolution, and other related issues: (3) Absence of laboratory tests essential for the assessment of liver function and complete blood, such as LSM, alanine aminotransferase (ALT), aspartate aminotransferase (AST), and platelet count; (4) poor medication adherence; and (5) loss to follow-up.

### Follow-up and LREs

All patients received antiviral therapy per standard guidelines [3], including entecavir, tenofovir, adefovir dipivoxil, and tenofovir dipivoxil. Follow-up began after the MRI, and only LREs occurring post-MRI were considered. All patients underwent MRI using a 3.0-T unit (Ingenia CX, Philips; Discovery 750 W, GE Healthcare) or a 1.5-T unit (SIGNA™ Creator, GE Healthcare). The index diagnostic test was an upper abdominal MRI with no reference standard diagnostic test. The primary endpoint was LRE development. Medical records of all enrolled patients were reviewed until July 2023 to identify LREs, including hepatorenal syndrome, ascites, hepatic encephalopathy, upper gastrointestinal variceal bleeding, and hepatocellular carcinoma. The last one was confirmed using at least two of the following radiological techniques: contrast-enhanced ultrasound, contrast-enhanced computed tomography, multi-sequence MRI, or gadolinium ethoxybenzyl diethylenetriaminepentaacetic acid-enhanced MRI [18].

#### Clinical data collection

Baseline demographic data (age, sex, weight, height, hypertension history, and alcoholism) and laboratory parameters (platelet count, aminotransferases, alpha-fetoprotein, albumin, and total bilirubin) were collected. LSMs conducted within six months before or after MRI were also included. We performed LSMs using transient elastography (Fibro Touch-C, Haisikaier). All procedures were performed by a skilled operator following a standard protocol. Patients fasted beforehand, and at least 10 valid measurements were obtained per patient. The operator recorded the average number of valid measurements, and only assessments meeting validity criteria were included in the analysis.

#### Image acquisition

All patients underwent an upper abdominal MRI. To extract imaging features, their images were retrieved from the picture archiving and communication system.

Two radiologists—Reader 1 (Y.K.L.) and Reader 2 (X.J.L.)—with 7 and 15 years of experience, respectively, assessed MRI image quality to determine eligibility for image segmentation and radiomics feature extraction. Both readers manually delineated the liver area on each cross-sectional MRI slice using ITK-SNAP software version 3.8 (www.itksnap.org). We carefully outlined the liver in each layer without exceeding its margin to minimize the potential impact on neighboring organs. Initially, images from 30 randomly selected patients were segmented by both readers. For cases with subjective errors, such as unclear liver edges due to respiratory artifacts, consensus was reached through discussion to ensure segmentation consistency. A week later, reader one repeated the procedure on the same 30 patients and segmented the remaining 192.

### Feature extraction and normalization

PyRadiomics, an open-source Python package (https://www.python.org/), was used to extract radiomics features from regions of interest. Extracted features included first-order features, Neighborhood Gray-Tone Difference Matrix, Gray Level Co-Occurrence Matrix, Gray Level Dependence Matrix, Gray Level Size Zone Matrix (GLSZM), Shape3D, Shape2D, Laplacian of Gaussian filter (kernel size: 4 × 5 pixels, height × width), and Wavelet-Based Features [19], totaling 1,050 features. We performed data cleaning and used a *t*-test to identify statistically significant features from the images (*p* < 0.05). Feature data were standardized using the *z*-score method to eliminate the influence of varying units and dimensions.

### Feature selection and radiomics model development

We first conducted intra-group assessments to evaluate the consistency of the same reader’s segmentations for the same patient at different time points, selecting features with strong reproducibility. Next, inter-group assessments measured segmentation consistency between readers for the same patient. Features were included in the analysis only if their intraclass correlation coefficients exceeded 0.75 in both assessments. Using computer-generated randomization, patients were assigned to training and test cohorts in a 7:3 ratio. To prevent overfitting, we used the least absolute shrinkage and selection operator (LASSO) algorithm to build a regression model, leveraging its strong dimensionality reduction capability to identify the top predictive features with nonzero LASSO coefficients. Eight machine learning algorithms, including logistic regression, support vector machine, random forest, extreme gradient boosting−XGBoost classifier, K-Nearest Neighbors, decision tree, extra trees, and stochastic gradient descent classifiers, were employed for feature selection and radiomics model development. The machine learning library Scikit-learn version 1.2.0 (https://scikit-learn.org/stable/) in Python (V3.7.0) was used to model and predict LREs based on quantitative radiomics features. The models’ inputs comprised features selected by LASSO, while the outputs represented follow-up results for each case. Using the specified characteristics, a 10-fold cross-validation of the training set validated the radiomics models’ effectiveness. The training process used a grid-search method to optimize hyperparameters for each model. Some representative hyperparameter combinations from the grid search are shown in Supplemental File 1 (Feature extraction). The area under the receiver operating characteristic curve (AUC) was analyzed to determine the most effective model.

### Validation and comparison of radiomics models

Model discrimination was assessed using a calibration plot. Calibration curves illustrate the alignment between model predictions and actual outcomes, offering an intuitive measure of reliability essential for gaining researchers’ and clinicians’ trust. Receiver operating characteristic curve analyses were also performed on the fibrosis-4 index, aspartate aminotransferase to platelet ratio index, and LSM, comparing these traditional clinical methods with our radiomics models. This comparison helps establish the role of MRI-based machine learning radiomics in predicting LREs.

### Machine learning explainable tool

The models were interpreted using SHapley Additive exPlanation−SHAP, which accurately quantifies each feature’s contribution to the final predictions [20]. These values allowed us to assess how each predictor positively or negatively influenced the target variable. Each radiomics feature was analyzed using its corresponding values. Additionally, the importance of permutation was applied to assess each feature’s significance in predicting outcomes. This method estimates the importance of observed feature distribution by iteratively permuting the result vector within a noninformative context [21].

### Statistical analyses

Descriptive statistics (means and standard deviations) were used for continuous variables with a normal distribution. Conversely, descriptive measures (median/interquartile range and minimum/maximum values) were used for continuous variables without a normal distribution. The AUC was calculated to assess model performance. All statistical tests were conducted as two-sided tests, with a significance threshold set at *p* < 0.05. All statistical analyses were performed using the R statistical package V4.1.2 (https://www.r-project.org/).

## Results

### Patient characteristics

**Fig. 1:**
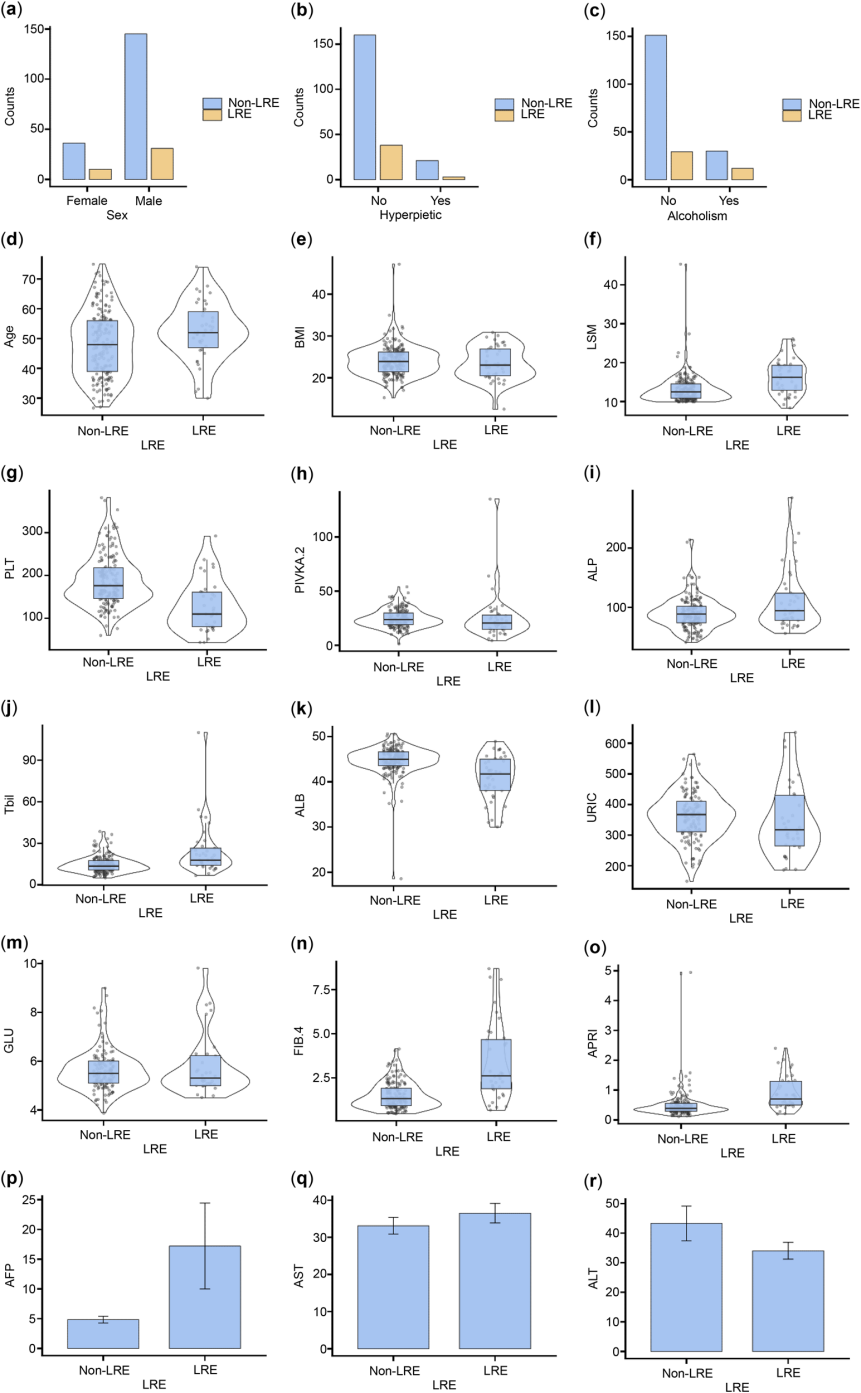
Baseline characteristics of the included patients. The figure compares demographic and clinical characteristics between the LRE and non-LRE groups. Data presentation: bar charts for categorical variables shown as counts (**a**–**c**); violin/box plots for continuous variables shown as median with interquartile range (IQR) (**d**–**o**); bar charts with error bars for continuous variables shown as mean with standard deviation (SD) (**p**–**r**). AFP, Alpha-fetoprotein; ALB, Albumin; ALP, Alkaline phosphatase; ALT, Alanine transaminase; APRI, Aminotransferase-to-platelet ratio index; AST, Aspartate aminotransferase; BMI, Body mass index; FIB-4, Fibrosis 4 score; GGT, Glutamyl transpeptidase; GLU, Fasting blood glucose; LRE, Liver-related event; LSM, Liver stiffness measurement; Non-LRE, Patients without liver-related event; PIVKA-2, Prothrombin induced by vitamin K absence or antagonist II; PLT, Platelet; Tbil, Total bilirubin; URIC, Serum uric acid

**Fig. 2:**
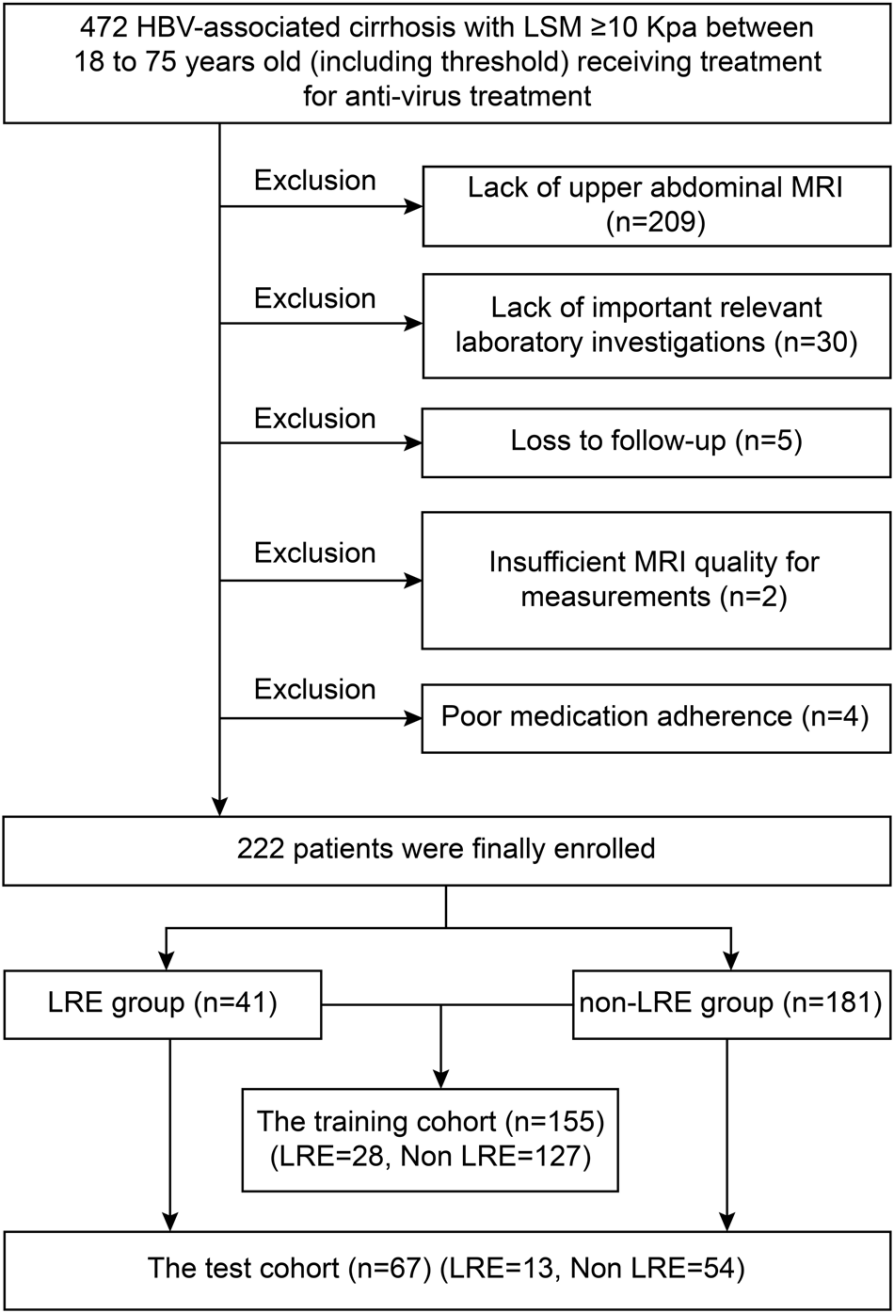
Flowchart showing the inclusion and exclusion of patients in this study. HBV, Hepatitis B virus; LRE, Liver-related event; LSM, Liver stiffness measurement; MRI, Magnetic resonance imaging

This study enrolled 222 patients aged 18 to 75 years with HBV-related fibrosis (LSM ≥ 10 kPa) who underwent antiviral treatment. LRE rates did not differ significantly between the training and test sets (Table 1). Similarly, no significant differences were observed in clinical indicators, including sex, age, platelet count, and LSMs, between the two groups. Fibrosis-4 scores and aspartate aminotransferase to platelet ratio index also showed no significant differences between the training and test cohorts (Table 2). Figure 1 shows the baseline characteristics of the included patients. The training and test sets comprised 155 and 67 patients, respectively, with 28 and 13 patients developing LREs (41 in total) (Fig. 2).

**Table 1:** Scanning parameters for magnetic resonance images

MRI scanner	Number	Sequence	TR/TE (ms)	Slice thickness (mm)	Slice gap (mm)	Field of view (mm)	Scanning matrix
3.0-T Ingenia CX, Philips	79	T1-w	3.22/0	4	2	448 × 448	268 × 265
		T2-w	2,725/86	6	7	432 × 432	296 × 258
		DWI	1,617/15	6	7	288 × 288	140 × 137
3.0-T Discovery 750 W, GE Healthcare	59	T1-w	4.54/1.67	5	2.5	512 × 512	256 × 200
		T2-w	6,000/79	6	7	512 × 512	320 × 320
		DWI	6,000/28	6	7	256 × 256	128 × 128
1.5-T SIGNA™ Creator, GE Healthcare	84	T1-w	6.16/3.13	5	2.5	256 × 256	256 × 192
		T2-w	3,157/82	6	7	512 × 512	320 × 320
		DWI	2,500/27	6	7	256 × 256	128 × 128

*MRI* Magnetic resonance imaging, *TR* Repetition time, *TE* Echo time, *DWI* Diffusion-weighted imaging, *T1-w* T1-weighted imaging, *T2-w* T2-weighted imaging

**Table 2:** Baseline characteristics of the 222 patients with a liver stiffness measurement (LSM) ≥ 10 kPa

Characteristic	Cohort			*p*-value^b^
	Overall *n* = 222^a^	Training *n* = 155^a^	Test *n* = 67^a^	
Age (years)	49 ± 11	49 ± 11	49 ± 10	0.933
Sex (*n*, %)				0.271
Female	46 (20.81)	29 (18.83)	17 (25.37)	
Male	175 (79.19)	125 (81.17)	50 (74.63)	
Alcoholism (*n*, %)				0.164
No	179 (81.00)	121 (78.57)	58 (86.57)	
Yes	42 (19.00)	33 (21.43)	9 (13.43)	
Hyperpietic (*n*, %)				0.284
No	197 (89.14)	135 (87.66)	62 (92.54)	
Yes	24 (10.86)	19 (12.34)	5 (7.46)	
Diabetes (*n*, %)				0.943
No	128 (80.50)	90 (80.36)	38 (80.85)	
Yes	31 (19.50)	22 (19.64)	9 (19.15)	
BMI (kg/m^2^)	24.0 ± 3.8	24.3 ± 4.1	23.3 ± 2.9	0.045
LSM (kPa)	13.70 ± 3.92	13.63 ± 4.14	13.80 ± 3.42	0.746
AFP (ng/mL)	7 ± 20	8 ± 24	5 ± 6	0.114
PIVKA-2 (mAu/mL)	25 ± 12	25 ± 13	25 ± 9	0.790
PLT (U/L)	178 ± 64	178 ± 65	178 ± 63	0.953
AST (U/L)	34 ± 27	35 ± 31	31 ± 15	0.187
ALT (U/L)	42 ± 70	45 ± 82	35 ± 23	0.183
ALP (U/L)	94 ± 33	95 ± 37	92 ± 24	0.570
Tbil (μmol/L)	17 ± 10	17 ± 11	15 ± 9	0.229
GGT (U/L)	45 ± 44	47 ± 49	39 ± 26	0.112
ALB (U/L)	44.1 ± 3.9	44.0 ± 4.2	44.2 ± 3.3	0.635
URIC (μmol/L)	358 ± 89	358 ± 92	359 ± 83	0.905
GLU (mmol/L)	5.66 ± 0.97	5.65 ± 1.03	5.68 ± 0.81	0.886
APRI	0.56 ± 0.48	0.59 ± 0.55	0.48 ± 0.27	0.083
FIB-4	1.85 ± 1.37	1.90 ± 1.47	1.74 ± 1.11	0.404

*AFP* Alpha-fetoprotein, *ALB* Albumin, *ALP* Alkaline phosphatase, *ALT* Alanine transaminase, *APRI* Aminotransferase-to-platelet ratio index, *AST* Aspartate aminotransferase, *BMI* Body mass index, *FIB-4* Fibrosis 4 score, *GGT* Glutamyl transpeptidase, *GLU* Fasting blood glucose, *LSM* Liver stiffness measurement, *PIVKA-2* Prothrombin induced by vitamin K absence or antagonist II, *PLT* Platelet, *Tbil* Total bilirubin, *URIC* Serum uric acid

^a^ Mean ± standard deviation or *n* (%)

^b^ Welch two-sample *t*-test; Pearson’s *χ*^2^ test

### Feature selection and model construction

**Fig. 3:**
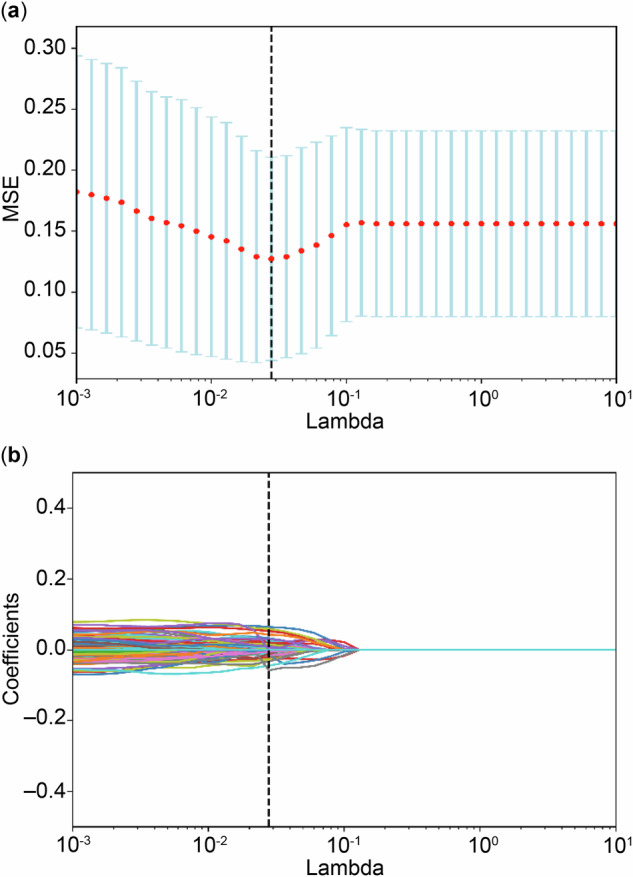
The least absolute shrinkage and selection operator (LASSO) algorithms. **a** LASSO regression feature screening chart, with dashed lines indicating the selected optimal log (*λ*) value of 0.02782559402207126, along with the location of one standard error, which generates the lowest possible mean squared error (MSE). **b** The LASSO convergence coefficient graph of the different omics features in the training group representing the curve of variation in image omics feature coefficients with log (*X*)

After the independent sample *t*-test and *z*-score standardization, 1,050 features were included in the LASSO regression. Figure 3 illustrates the feature selection process for predicting LREs. Ultimately, 58 features were selected for further analysis. Among them, Wavelet-LHH Gray-Level Run Length Matrix (GLRLM) Gray-Level Non-Uniformity Normalized from diffusion-weighted imaging (DWI) had the highest absolute regression coefficient, making it the most influential feature in enhancing predictive performance.

**Fig. 4:**
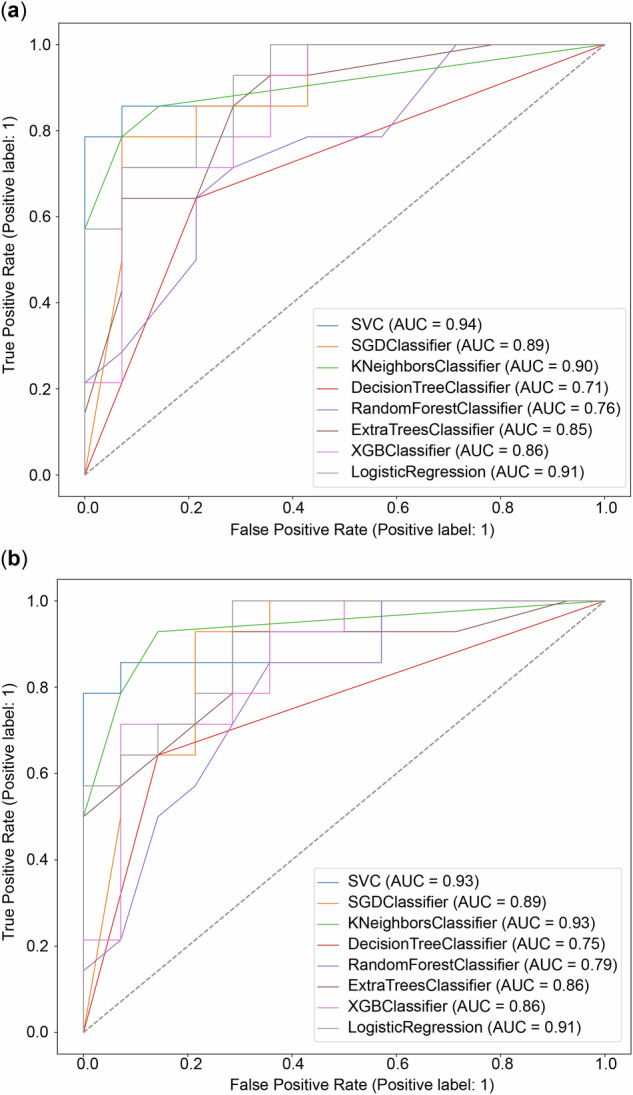
Receiver operating characteristic (ROC) curves and the corresponding calibrations. **a** ROC curves of the radiomics models from different methods for the training cohort. **b** ROC curves of the radiomics models from different methods for the test cohort. SGD, Stochastic gradient descent; SVC*,* Support vector machine classifier; AUC*,* Area under the curve; XGB*,* Extreme gradient boosting

Using the top 58 features identified in the selection process, eight radiomics models were developed with corresponding classifiers to predict LREs in the training and test sets (Fig. 4a, b). The support vector machine classifier radiomics model exhibited superior predictive performance, achieving AUCs of 0.94 and 0.93 in the training and test sets, respectively, outperforming all other classifiers. The model details and the corresponding coefficient weight map of the features are shown in Supplemental File 2 (Table S1 and Fig. S1). Patients with LREs had significantly higher radiomics scores than those without LREs across the training, test, and overall cohorts (Supplemental Fig. S2).

### Clinical application and apparent performance of the model

**Fig. 5:**
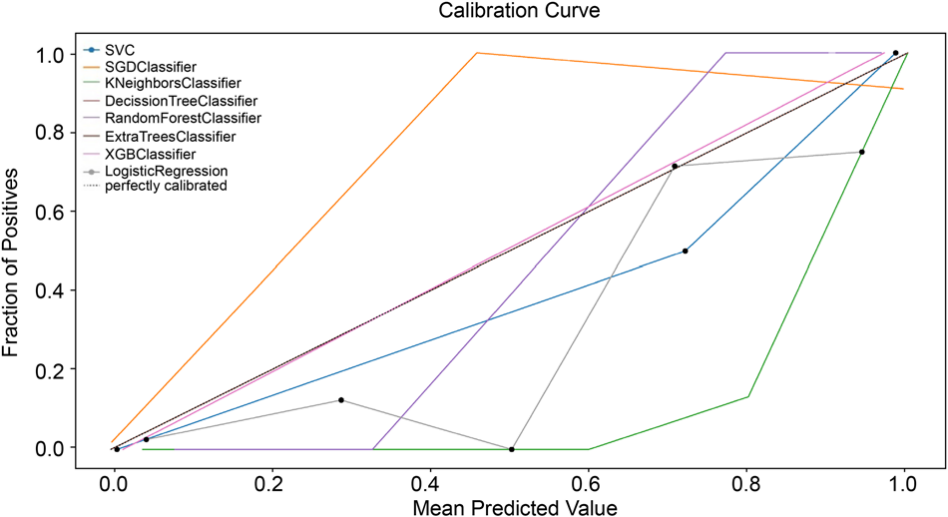
Calibration curves of the radiomics models from different methods for the whole cohort. SGD, Stochastic gradient descent; SVC*,* Support vector machine classifier; XGB*,* Extreme gradient boosting

The calibration curves of the eight radiomics models illustrate the agreement between predictions and observations, showing some deviation from a perfect fit (Fig. 5). The AUCs of traditional clinical tools are presented in Supplemental Fig. S3. The overall AUC of the radiomics models exceeded that of traditional clinical tools, highlighting their clinical utility and effectiveness. Relative confusion matrices and detailed model performance metrics, including accuracy, specificity, recall, precision, and F1 score, are provided in Supplemental Fig. S4.

### Explanation of model prediction

**Fig. 6:**
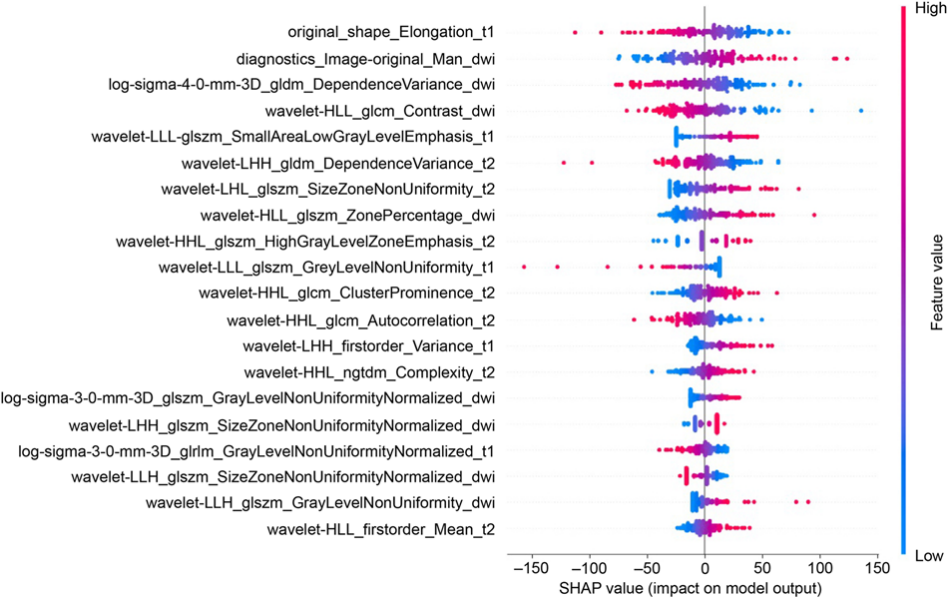
SHAP values of the top 20 radiomics features as predictors of liver-related events. *SHAP,* SHapley Additive explanation

Permutation importance results were ranked in descending order based on feature importance scores (Supplemental Fig. S5) to identify the most influential features in model predictions, aiding in understanding how the model identifies key predictors. The average log-sigma-3-0-mm-3D GLSZM Gray-Level Non-Uniformity Normalized in the DWI exhibited the highest predictive value across all prediction horizons. Additionally, SHapley Additive exPlanation values were used to determine the positive and negative relationships between the radiomics signature and LRE outcomes. In Fig. 6, the horizontal position indicates whether a specific value contributes to a higher or lower prediction. At the same time, the color represents whether the variable is high (red) or low (blue) for that observation.

## Discussion

Oral antiviral therapy is increasingly recognized as a standard treatment for delaying HBV-related fibrosis and cirrhosis. However, some patients still develop LREs, such as hepatocellular carcinoma or hepatic decompensation. Previous predictive studies on HBV-related fibrosis have primarily focused on clinical features, while the potential of radiological imaging as a noninvasive method to capture the spatial heterogeneity of liver disease remains underexplored. Remarkably, efforts to identify fibrosis features that predict responses to antiviral treatments have largely overlooked this issue [22, 23]. The heterogeneity of the liver microenvironment may contribute to variability in treatment response. Therefore, we aimed to predict LRE risk in liver fibrosis using machine learning radiomics methods based on multi-sequence MRI of the whole liver.

To our knowledge, this is the first study to predict LREs in patients with chronic HBV-associated fibrosis using an MRI-based radiomics approach. This method offered a more comprehensive assessment than computed tomography- or ultrasound-based radiomics [24, 25] and significantly improved prediction accuracy with multi-sequence MRI. We assessed the impact of computational dimensionality—two-dimensional (2D) slice-wise *versus* three-dimensional (3D) volumetric feature extraction—using identical 3D volume of interest masks for both approaches. This design isolates computational methodology effects from volume of interest spatial variations, enabling a direct comparison of texture characterization capabilities.

MRI-based quantitative radiomics exhibited strong performance as a noninvasive, high-throughput approach for predicting LREs in patients with HBV-related fibrosis. The support vector machine classifier achieved AUCs of 0.94 and 0.93 in the training and test sets, respectively. Support vector machine has been recognized as highly effective for radiomics analysis and is well-suited for this classification task [26, 27]. The AUCs of the remaining seven machine learning algorithms were all ≥ 0.71 in both cohorts, with most exceeding 0.85. While fibrosis-4 index, aspartate aminotransferase to platelet ratio index, and LSM are widely used clinical tools, our radiomics models demonstrated superior AUCs. Overall, our findings suggest that machine learning can effectively predict LREs in patients with fibrosis and chronic hepatitis B, demonstrating comparable accuracy across algorithms and underscoring radiology’s potential to enhance clinical decision-making. This approach enables identifying patients who require more frequent follow-ups and extended therapy.

Liver function deteriorates at the cellular level due to damage or fibrosis, often associated with low-grade inflammation [28, 29]. Radiomics features capture subtle liver changes, offering a more precise representation of the complex physiopathological processes underlying LREs. Although MRI provides limited evidence of significant macroscopic liver alterations, fibrosis progression may lead to liver darkening, reduced blood flow, and decreased volume [30, 31]. Our models leverage these changes to predict LREs.

Fifty-eight radiomics features were extracted from liver MRI scans. Some were linked to liver shape (*e.g.,* original-shape elongation), wavelets (*e.g.,* Wavelet-HLL Gray Level Co-Occurrence Matrix Contrast), and texture (*e.g.,* Gray-Level Non-Uniformity Normalized), all essential for predicting LREs. Notably, lower original-shape elongation values indicate rough, uneven liver edges. This aligns with prior research showing that fibrosis exacerbates liver fissures and shrinkage, impairing detoxification and compensatory function and ultimately increasing LRE risk [32]. Gray-level non-uniformity from GLSZM reflects grayscale heterogeneity in liver tissue, likely resulting from inflammation, fibrosis, nodules, and micronecrosis, all contributing to LRE development [33]. Wavelet features are closely linked to cell morphology, pathophysiology, and proteomics. Notably, most of our study’s final selected radiomics features were wavelet-based, aligning with findings from other radiomics research [34–36]. Overall, our MRI-based radiomics models exhibited strong predictive potential for improving outcomes in individuals with HBV-associated fibrosis. Our findings highlight the feasibility and clinical value of radiomics models in medical imaging analysis for predicting disease outcomes in chronic liver disease. We recommend a more aggressive monitoring and treatment strategy for high-risk patients identified on baseline MRI radiomics. This approach may involve increasing follow-up frequency, shortening intervals for high-risk patients to closely monitor disease progression for early diagnosis and screening of LREs. Extending the treatment cycle is another strategy, allowing longer treatment durations or dose adjustments within guideline recommendations to prevent LREs more effectively. Additionally, enhancing patient education for high-risk individuals can improve understanding of their condition and the importance of adherence, thereby boosting treatment compliance.

This study has limitations. First, its retrospective, single-center design may restrict the generalizability of our models. External validation, a larger sample size, and prospective studies are necessary to establish a more reliable predictive model. Second, while machine learning models can predict medical outcomes, data availability and accessibility must be carefully assessed, as they directly impact clinical applicability. This study’s manual liver segmentation ensured accuracy but required substantial time and effort. In the future, greater emphasis should be placed on semi-automatic and automatic liver tissue segmentation methods. Third, the imaging-based model was not integrated with a clinical indicator model. This limitation may have hindered our understanding of the relationship between imaging findings and clinical outcomes, potentially reducing the model’s overall effectiveness in predicting and monitoring patient outcomes. Future studies integrating imaging biomarker models with clinical indicators could enhance prognostic accuracy.

In conclusion, we developed a radiomics model using liver MRI, which may enhance risk prediction and management in patients with HBV-associated chronic fibrosis. This model offers valuable prognostic insights and supports clinical decision-making. Our model may help predict LRE risk in this patient population. Further research and validation are needed to confirm the clinical utility of this radiomics approach in managing HBV-associated chronic fibrosis.

Patient data cannot be publicly shared due to privacy concerns, but may be obtained from the corresponding author upon reasonable request, subject to approval by the institutional review board of Zhuhai People’s Hospital (xwg315@163.com).

## Supplementary information

**Additional file 1: Fig. S1.** Feature coefficient weight map. **Supplemental Fig. S2.** Box-and-whisker plots and waterfall plots of the radiomics score (Rad score) derived from the Least Absolute Shrinkage and Selection Operator (LASSO) feature for predicting liver-related events (LREs) in hepatitis B virus (HBV)-associated cirrhosis patients receiving oral antivirals. A, D: Entire primary cohort. B, E: Training cohort. C, F: Test cohort. The label ‘0’ indicates patients without LRE, whereas the label ‘1’ represents patients with LRE. **Supplemental Fig. S3.** Receiver operating characteristic curve analysis of Fibrosis 4 Score (FIB-4), aminotransferase-to-platelet ratio index (APRI), and liver stiffness measurement (LSM). **Supplemental Fig. S4.** Relative confusion matrices of the models in the training cohort (A) and test cohort (B). Model performances in the training cohort (C) and test cohort (D). **Supplemental Fig. S5.** Weights of radiomics signature importance.

## Data Availability

Patient data cannot be publicly shared due to privacy concerns, but may be obtained from the corresponding author upon reasonable request, subject to approval by the institutional review board of Zhuhai People’s Hospital (xwg315@163.com).

## Abbreviations

3D: Three-dimensional
AUC: Area under the receiver operating characteristic curve
DWI: Diffusion-weighted imaging
GLSZM: Gray Level Size Zone Matrix
HBV: Hepatitis B virus
LASSO: Least absolute shrinkage and selection operator
LRE: Liver-related event
LSM: Liver stiffness measurement
MRI: Magnetic resonance imaging
