# Supplementary material for: MRI-based machine-learning radiomics of the liver to predict liver-related events in hepatitis B virus-associated fibrosis
Source: Eur Radiol Exp. 2025 Aug 27;9:81. doi: 10.1186/s41747-025-00602-0 (PMC12390902; doi:10.1186/s41747-025-00602-0)
Supplement: Supplementary file 1 — Additional file 1: Fig. S1. Feature coefficient weight map. Supplemental Fig. S2. Box-and-whisker plots and waterfall plots of the radiomics score (Rad score) derived from the Least Absolute Shrinkage and Selection Operator (LASSO) feature for predicting liver-related events (LREs) in hepatitis B virus (HBV)-associated cirrhosis patients receiving oral antivirals. A, D: Entire primary cohort. B, E: Training cohort. C, F: Test cohort. The label ‘0’ indicates patients without LRE, whereas the label ‘1’ represents patients with LRE. Supplemental Fig. S3. Receiver operating characteristic curve analysis of Fibrosis 4 Score (FIB-4), aminotransferase-to-platelet ratio index (APRI), and liver stiffness measurement (LSM). Supplemental Fig. S4. Relative confusion matrices of the models in the training cohort (A) and test cohort (B). Model performances in the training cohort (C) and test cohort (D). Supplemental Fig. S5. Weights of radiomics signature importance. [file 41747_2025_602_MOESM1_ESM.pdf]

MRI-based machine-learning radiomics of the liver to predict liver-related events in hepatitis B virus-associated fibrosis

ELECTRONIC SUPPLEMENTARY MATERIAL

Supplemental File 1. Feature extraction

The steps below were undertaken for feature extraction following manual tumor segmentation:

The bin width of the gray histogram was set to 25 to balance the fine-grained information of texture features with computational efficiency. To avoid features being affected by differences in resolution, normalization was then performed before feature extraction. Value plausibility was ascertained for all images at initial assessment. Images were spatially resampled to a 3x3x3 mm voxel size using the Nearest Neighbor interpolator in ITK-SNAP version 3.8. No re-segmentation was performed, as the original segmentation was deemed sufficiently accurate for the analysis, minimizing potential bias introduced by manual intervention. Radiomic features were extracted using the PyRadiomics package (developed by the Radiomics team). Original image type including first order statistics, 3D shape-based, 2D shape based, Gray Level Cooccurence Matrix, Gray Level Run Length Matrix, Gray Level Size Zone Matrix, Neighboring Gray Tone Difference Matrix and Gray Level Dependence Matrix features as well as Laplacian of Gaussian-filtered and wavelet-based filtered versions of these features. Laplacian of Gaussian-filtering was performed with a kernel (*sigma)* of 3mm or 4mm. Gray Level Co-Occurrence Matrix and GLRLM were extracted using the default settings (separately for each direction then averaged). Feature descriptions can be found in the PyRadiomics documentation (https://pyradiomics.readthedocs.io/en/latest/features.html).

Grid Search

The following process was performed using the Python programming language version 3.7.

Feature engineering was carried out by fitting the sklearn.preprocessing StandardScaler method with feature_range=(0,1) to the training data only and transforming the training and test data with the fitted scaler.

Scikit Learn offers a wide range of model options, such as logistic regression, decision trees, random forests. For assessing the optimal hyperparameters, a grid search was performed over the following hyperparameter settings:

```
param_grid_svm = {
    'C': [0.1, 1, 10, 100],
    'kernel': ['linear', 'poly', 'rbf', 'sigmoid'],
    'gamma': ['scale', 'auto', 0.01, 0.1, 1, 10],
    'degree': [2, 3, 4],
    'coef0': [0.0, 0.5, 1.0]
}
svm_model = svm.SVC()
grid_search_svm = GridSearchCV(svm_model, param_grid_svm, cv=10)

param_grid_sgd = {
'loss': ['hinge', 'log'],
    'penalty': ['l1', 'l2', 'elasticnet'],
    'alpha': [0.0001, 0.001, 0.01, 0.1],
    'fit_intercept': [True, False],
    'max_iter': [100, 200, 300],
    'tol': [1e-3, 1e-4],
    'learning_rate': ['constant', 'optimal', 'invscaling'],
    'eta0': [0.01, 0.1, 0.5]
}
sgd_model = linear_model.SGDClassifier()
```

```
grid_search_sgd = GridSearchCV(sgd_model, param_grid_sgd, cv=10)

param_grid_knn = {
    'n_neighbors': [3, 5, 7, 9, 11, 13],
    'weights': ['uniform', 'distance'],
    'algorithm': ['auto', 'ball_tree', 'kd_tree', 'brute'],
    'leaf_size': [10, 20, 30, 40],
    'p': [1, 2],
    'metric': ['euclidean', 'manhattan', 'minkowski']
}
knn_model = neighbors.KNeighborsClassifier()
grid_search_knn = GridSearchCV(knn_model, param_grid_knn, cv=10)

param_grid_dt = {
    'criterion': ['gini', 'entropy'],
    'splitter': ['best', 'random'],
    'max_depth': [None, 10, 20, 30, 40, 50],
    'min_samples_split': [2, 5, 10, 20],
    'min_samples_leaf': [1, 2, 4, 6],
    'max_features': ['auto', 'sqrt', 'log2', 5],
    'max_leaf_nodes': [None, 10, 20, 30],
    'min_impurity_decrease': [0.0, 0.01, 0.1]
}
dt_model = tree.DecisionTreeClassifier()
grid_search_dt = GridSearchCV(dt_model, param_grid_dt, cv=10)

param_grid_rf = {
    'n_estimators': [100, 200, 300, 400, 500],
    'max_depth': [None, 10, 20, 30, 40, 50],
    'min_samples_split': [2, 5, 10, 20],
```

```
        'min_samples_leaf': [1, 2, 4, 6],
        'max_features': ['auto', 'sqrt', 'log2', 5],
        'criterion': ['gini', 'entropy'],
        'bootstrap': [True, False]
    }
    rf_model = ensemble.RandomForestClassifier()
    grid_search_rf = GridSearchCV(rf_model, param_grid_rf, cv=10)

    param_grid_xgb = {
       'n_estimators': [100, 200, 300],
        'max_depth': [3, 4, 5, 6, 7],
        'learning_rate': [0.01, 0.05, 0.1, 0.2],
        'min_child_weight': [1, 3, 5],
        'gamma': [0, 0.1, 0.2],
        'subsample': [0.7, 0.8, 0.9],
        'colsample_bytree': [0.7, 0.8, 0.9],
        'reg_alpha': [0, 0.1, 0.5],
        'reg_lambda': [1, 1.5, 2]
    }
    xgb_model = xgboost.XGBClassifier(use_label_encoder=False,
eval_metric='logloss')
    grid_search_xgb = GridSearchCV(xgb_model, param_grid_xgb, cv=10)


    param_grid_et = {
        'n_estimators': [100, 200, 300, 400, 500],
        'max_depth': [None, 10, 20, 30, 40, 50],
        'min_samples_split': [2, 5, 10, 20],
        'min_samples_leaf': [1, 2, 4, 6],
        'max_features': ['auto', 'sqrt', 'log2', 5],
```

```
    'criterion': ['gini', 'entropy'],
    'bootstrap': [True, False],
    'max_leaf_nodes': [None, 10, 20, 30]
}
et_model = ensemble.ExtraTreesClassifier()
grid_search_et = GridSearchCV(et_model, param_grid_et, cv=10)

param_grid_lr = {
    'C': [0.1, 1, 10],
    'penalty': ['l1', 'l2']
}
lr_model = LogisticRegression()
grid_search_lr = GridSearchCV(lr_model, param_grid_lr, cv=10)
```

Supplemental File 2. Details of the best model (Table S1) and features coefficient weight map (Fig. S1).

Table S1

Sequence	Feature	Coefficient
**T1-w**	original shape Elongation	-0.3591714709988225
	original GLSZM Large Area Emphasis	2.0144625574582364e–09
	log-sigma-3-0-mm-3D GLRLM Gray Level Non-Uniformity Normalized	–0.5385843154763131
	wavelet-LHH First Order Energy	8.464989860066501e–05
	wavelet-LHH First Order Mean	23.650465027053773
	wavelet-LHH First Order Total Energy	2.372396446300982e–06
	wavelet-LHH First Order Variance	2.891623806074527
	wavelet-HLH First Order Median	-6.478718147949892
	wavelet-HLH First Order Minimum	-0.020431922282140712
	wavelet-HLH GLSZM Size Zone Non-Uniformity	-0.018675333527418476
	wavelet-HLH GLSZM Zone Entropy	-0.03629899798092863
	wavelet-HHH First Order Skewness	-0.0062773364220801466
	wavelet-HHH GLSZM Small Area Low Gray Level Emphasis	-0.07763728485041886
	wavelet-LLL First Order 90Percentile	-0.02159137576114733
	wavelet-LLL First Order Root Mean Squared	-0.012990888704074253
	wavelet-LLL GLRLM Long Run Emphasis	-0.0001509695915447784

	wavelet-LLL GLSZM Gray Level Non-Uniformity	0.002947559982881934
	wavelet-LLL GLSZM Small Area Low Gray Level Emphasis	0.036309341315023606
**T2-w**	diagnostics Image-interpolated Minimum	-0.0051567139292610234
	log-sigma-3-0-mm-3D GLSZM Size Zone Non-Uniformity	-0.0017937096355111553
	wavelet-LLH GLCM Cluster Tendency	-0.6332164452016459
	wavelet-LHL GLSZM Gray Level Non-Uniformity	5.526378496974897e–05
	wavelet-LHL GLSZM Size Zone Non-Uniformity	0.016259407528495855
	wavelet-LHL GLSZM Small Area Low Gray Level Emphasis	0.055171290448667115
	wavelet-LHH GLDM Dependence Variance	-0.0034004050588922275
	wavelet-LHH GLSZM Gray Level Non-Uniformity	-0.0038364164393160215
	wavelet-HLL First Order Mean	2.399018513946315
	wavelet-HLL GLRLM Gray Level Non-Uniformity Normalized	115.70851303706205
	wavelet-HLL GLRLM Gray Level Variance	-2.4185782769845556e–07
	wavelet-HLL GLRLM Short Run Low Gray Level Emphasis	-2.099850012465584

	wavelet-HLL GLSZM Small Area Low Gray Level Emphasis	0.21833806451527848
	wavelet-HLH First Order Median	19.443898345230025
	wavelet-HLH GLSZM Gray Level Non-Uniformity Normalized	-0.15402286470776613
	wavelet-HLH GLSZM Gray Level Variance	4.380749432016191e–13
	wavelet-HHL GLCM Autocorrelation	1.438190915046489
	wavelet-HHL GLCM Cluster Prominence	1.4816002830141106
	wavelet-HHL GLSZM High Gray Level Zone Emphasis	0.010031974711137977
	wavelet-HHL GLSZM Zone Entropy	0.027276816414752686
	wavelet-HHL NGIDM Complexity	-0.845870218600511
	wavelet-LLL First Order Kurtosis	-0.0007003560501224118
**DWI**	diagnostics Image-original Mean	0.003095542823107821
	original shape Maximum 2D-Diameter Slice	-0.000472391561326937
	log-sigma-3-0-mm-3D GLSZM Gray Level Non-Uniformity Normalized	0.15275824407390576
	log-sigma-4-0-mm-3D First Order Energy	-5.0686715112416364e–06
	log-sigma-4-0-mm-3D GLDM Dependence Variance	0.009741537803321536
	wavelet-LLH GLRLM Run Variance	0.01649310037789892

wavelet-LLH GLSZM Gray Level Non-Uniformity	0.022455603309570766
wavelet-LLH GLSZM Size Zone Non-Uniformity Normalized	-0.03368790601705106
wavelet-LHH GLRLM Gray Level Non-Uniformity Normalized	1018.7432079898336
wavelet-LHH GLSZM Size Zone Non-Uniformity Normalized	0.6156681105867566
wavelet-HLL First Order Skewness	-0.0344016758219794
wavelet-HLL GLCM Contrast	-5.874716676359653
wavelet-HLL GLSZM Zone Percentage	154.69129324942088
wavelet-HLH GLRLM High Gray Level Run Emphasis	-9.364490897820948
wavelet-HHL GLRLM High Gray Level Run Emphasis	2.024823045211711
-wavelet-HHL GLSZM High Gray Level Zone Emphasis	-0.010545720834291087
wavelet-HHL GLSZM Low Gray Level Zone Emphasis	9.69413793142753e–15
wavelet-HHH GLSZM Size Zone Non-Uniformity	0.038970754749196944

T1-w, T1-weighted, T2-w, T2-weighted, DWI, diffusion-weighted imaging

Fig. S1. Features coefficient weight map

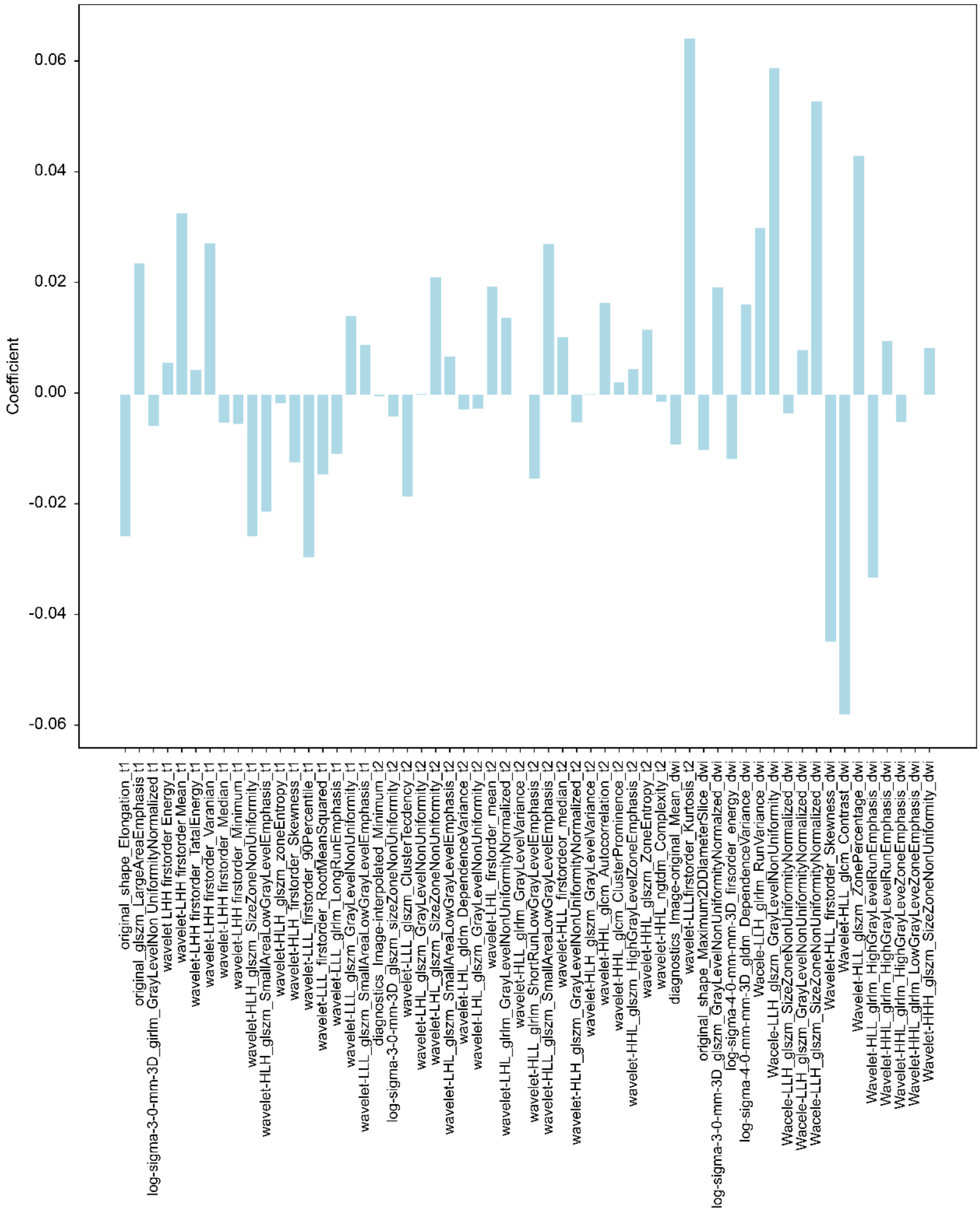

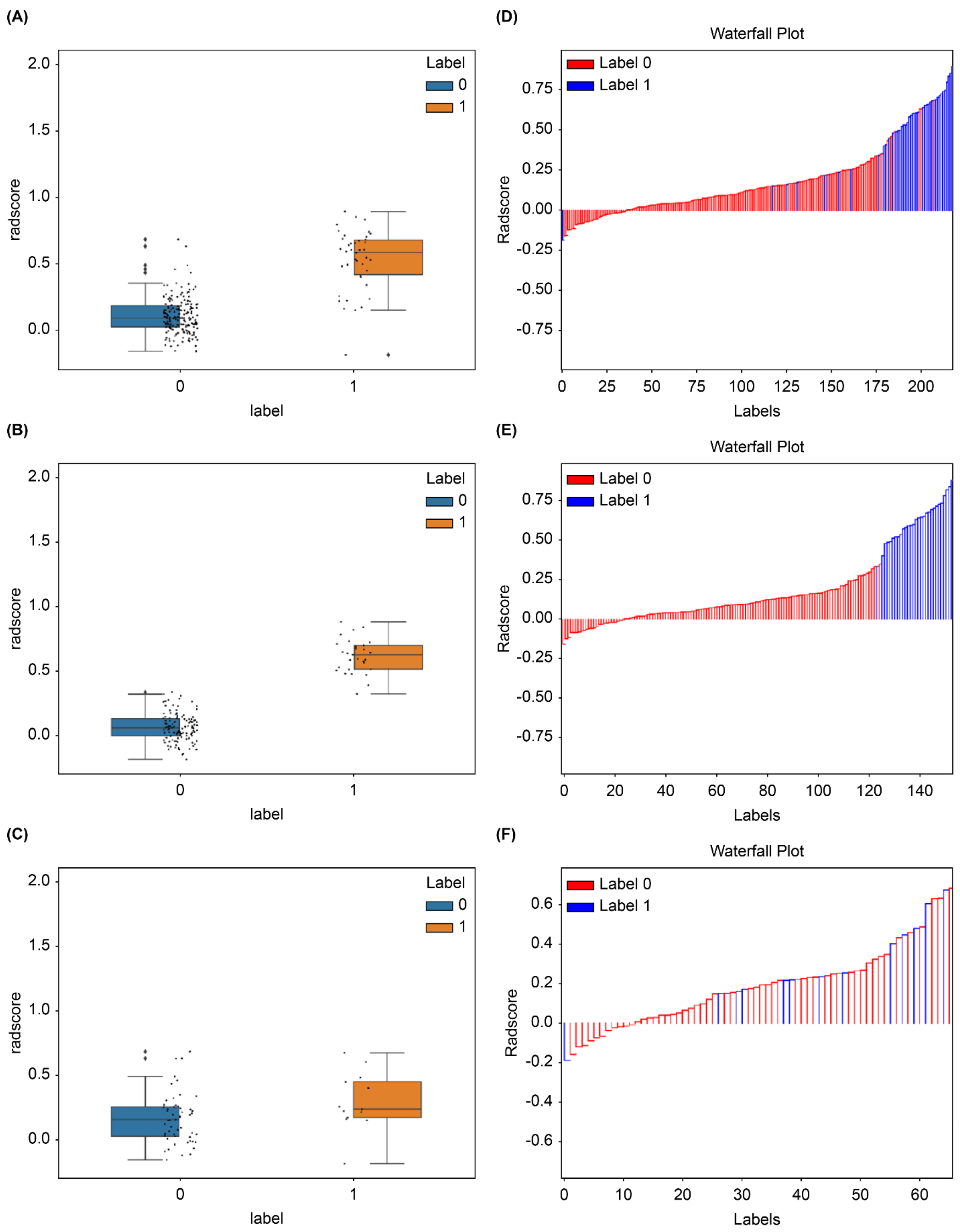

Supplemental Fig. S2. Box-and-whisker plots and waterfall plots of the radiomics score (Rad score) derived from the Least Absolute Shrinkage and Selection Operator (LASSO) feature for predicting liver-related events (LREs) in hepatitis B virus (HBV)-associated cirrhosis patients receiving oral antivirals. A, D: Entire primary cohort. B, E: Training cohort. C, F: Test cohort. The label ‘0’ indicates patients without LRE, whereas the label ‘1’ represents patients with LRE.

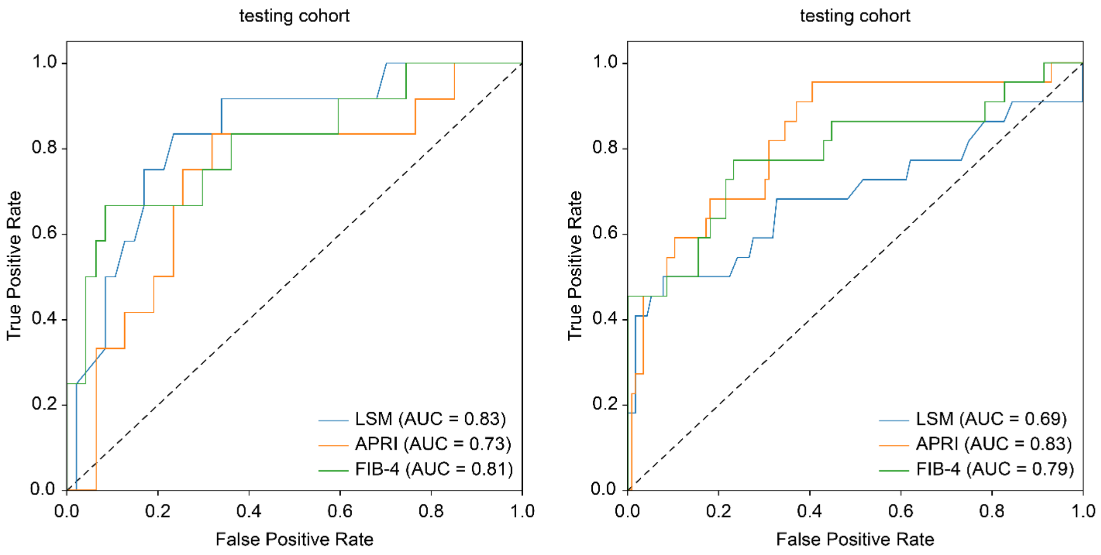

Supplemental Fig. S3. Receiver operating characteristic curve analysis of Fibrosis 4 Score (FIB-4), aminotransferase-to-platelet ratio index (APRI), and liver stiffness measurement (LSM).

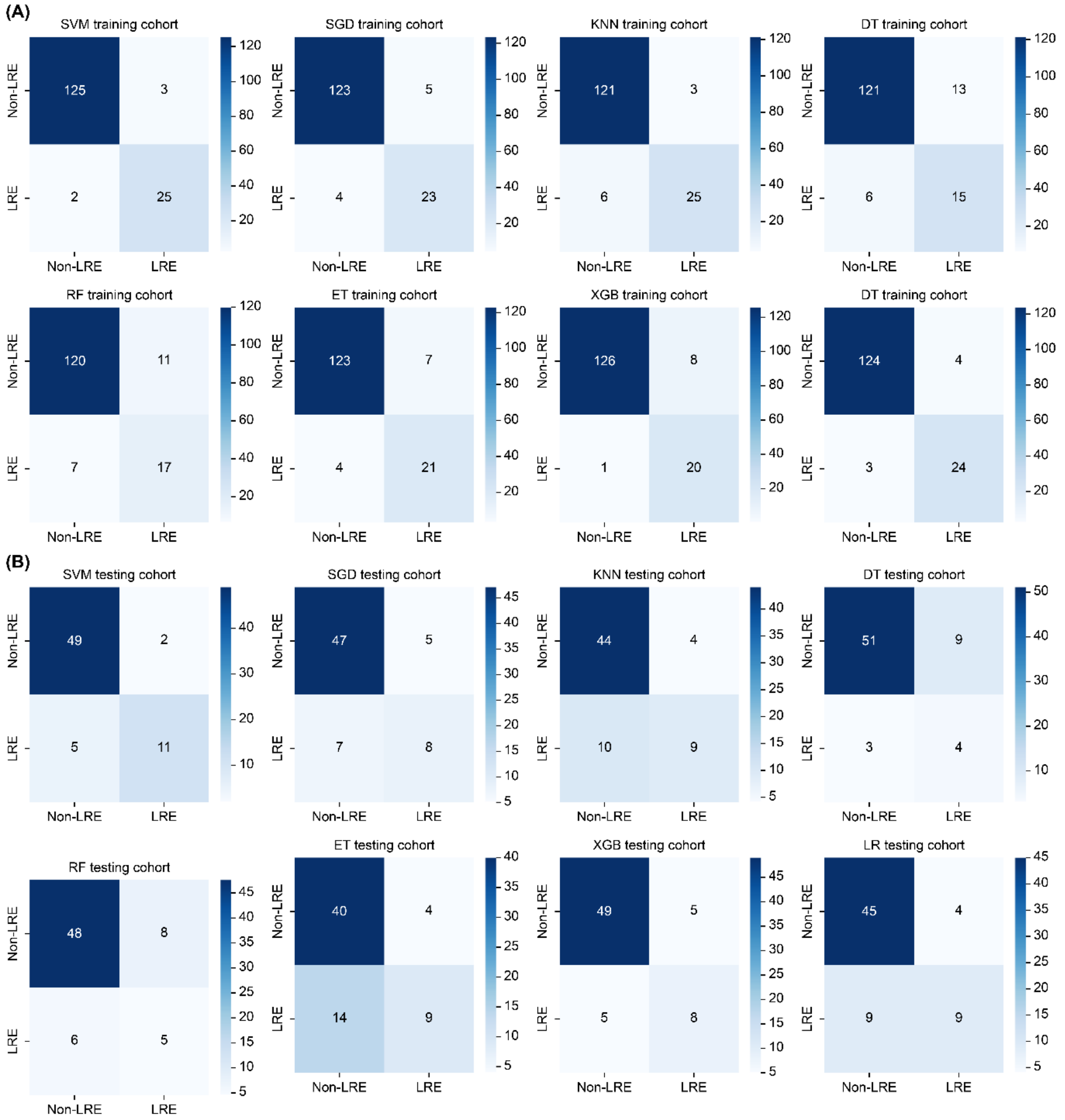

(C)

Model	Accuracy	Specificity	Recall	Precision	F1_score
SVM	0.967742	0.976563	0.925926	0.892857	0.925212
SGD	0.941935	0.960938	0.851852	0.821429	0.76412
KNN	0.941935	0.975806	0.806452	0.892857	0.91638
DT	0.877419	0.902985	0.714286	0.535714	0.687679
RF	0.883871	0.916031	0.708333	0.607143	0.722789
ET	0.929032	0.946154	0.84	0.75	0.785047
XGB	0.941935	0.940299	0.952381	0.714286	0.809249
LR	0.954839	0.96875	0.888889	0.857143	0.775665

(D)

Model	Accuracy	Specificity	Recall	Precision	F1_score
SVM	0.895522	0.960784	0.6875	0.846154	0.758621
SGD	0.820896	0.903846	0.533333	0.615385	0.551724
KNN	0.791045	0.916667	0.473684	0.692308	0.5625
DT	0.820896	0.85	0.571429	0.307692	0.4
RF	0.791045	0.857143	0.454545	0.384615	0.451128
ET	0.731343	0.909091	0.391304	0.692308	0.54878
XGB	0.850746	0.907407	0.615385	0.615385	0.59893
LR	0.80597	0.918367	0.5	0.692308	0.545455

Supplemental Fig. S4. Relative confusion matrices of the models in the training cohort (A) and test cohort (B). Model performances in the training cohort (C) and test cohort (D).

SVM, support vector machine, SGD, stochastic gradient descent, KNN, K-nearest neighbors, DT, Decision tree, RF Random Forest, ET, Extremely randomized trees, XGB, extreme gradient boosting, LR, Logistics regression

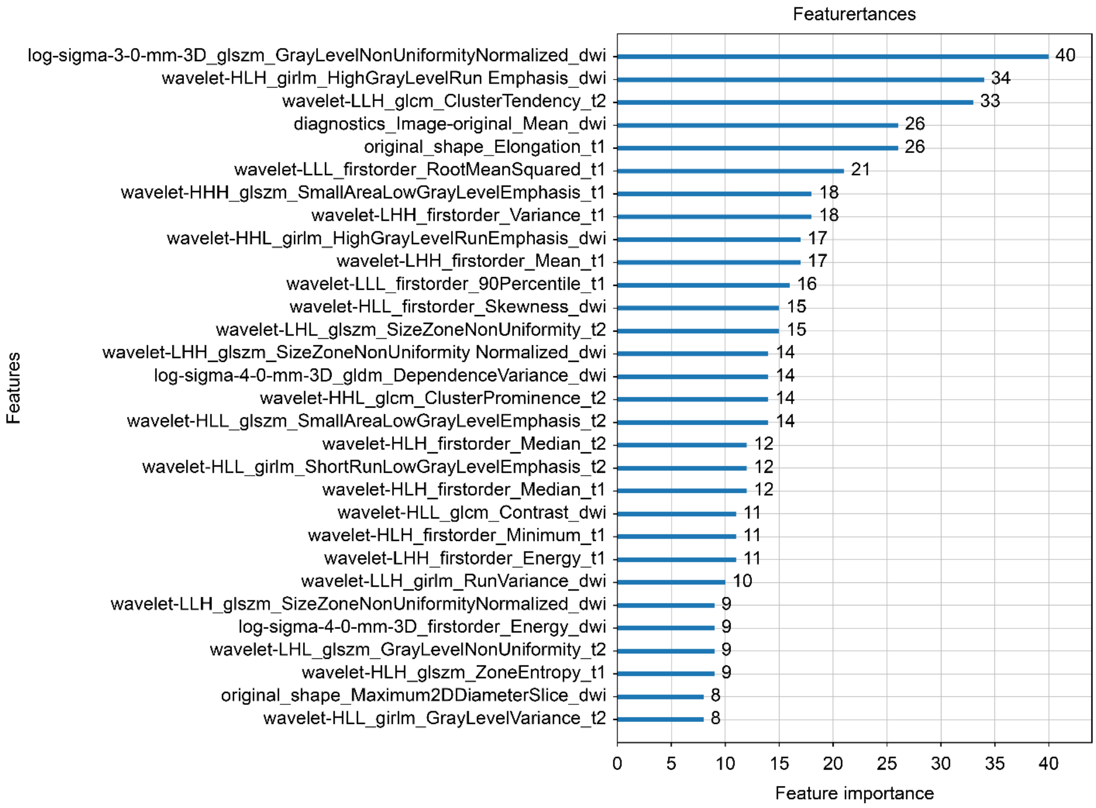

Supplemental Fig. S5 Weights of radiomics signature importance
